# The Platonic Receptacle (*Hypodoché*), Whitehead’s Philosophy, and Genome Evolution

**DOI:** 10.3390/v9120381

**Published:** 2017-12-14

**Authors:** Jan Svoboda, Jan Svoboda

**Affiliations:** 1 Czech Academy of Sciences, Institute of Molecular Genetics, Videnska 1083, CZ-14220 Prague 4, Czech Republic; jansva@seznam.cz; 2 Czech Academy of Sciences, Institute of Philosophy, Jilska 1, CZ-11000 Prague 1, Czech Republic

**Keywords:** Platonic receptacle, Whitehead’s argument, realization of ideas, present day genetics, genetic code, evolution changes

## Abstract

The discovery of a universal genetic code utilized by all existing organisms became the backbone of biology. The coding capacity underwent changes during evolution, but its main fluctuation results from its different reading and regulation. The genetic code thus represents a sort of receptacle of living organism evolution. In this article, we propose an analogy between the genetic code and a broader Platonic *hypodoché*, a concept that Alfred North Whitehead used to explain various aspects of science.

The present array of natural sciences has been inspired by the Platonic concept of ideas and Aristotelian critical questioning, classification of the known observations and their interpretation included. The strong impact of Plato’s idea formulation on modern science was revisited by Alfred North Whitehead, who underlined that new scientific institutions are in fact based on the realization of a particular idea.

In this article, we concentrate on the significance of Plato’s postulate of *hypodoché* (receptacle) that he proposed in the dialogue *Timaeus* [1]. He questioned Empedocles’ postulation of four elements (earth, fire, air, and water). Plato accepted their role in the creation of the cosmos but ascribed to these elements the role of modifiers. He introduced three principal factors to cosmology: The ideal model that can be apprehended by reason and its visible imitation. The third one (*triton allo genos*) [1] he called the receptacle (more generally, “nurse”) of all that had arisen. He characterized the receptacle as an invisible and formless all-accepting thing that belongs to the world of reason, but understanding it represents a very hard task. Such a receptacle is influenced by the four elements, but reciprocally imparts its activity upon them.

Even though the *hypodoché* is originally completely indefinite, ideas create a kind of action in it, leading to its (inner) concretization and differentiation, and in this special, indefinite way the *hypodoché* gives the space-time world its basic shapeability: it is what can take shape (*ekmageion*) [1]. In relation to the sensory world, this residual X represents itself as a potentiality, which, with its typical manner or specific style, gives reality to the ephemeral phenomena of this visible world.

Whitehead also points out that for all the variability of phenomena, it is necessary to presuppose the reality of a permanent unifying principle [2] that cannot be identified with divinity, with the human soul, or even with ideas in the original Platonic sense [3]. The *hypodoché* is formless and can take on any form, and because it is capable of infinite variation, its own (eternal) potency stands far beyond any space-time determination; its original nature merely suggests in principle a certain, very specific, yet indefinite relation to this determination. It is revealed as the influence of necessity and hence as the pure possibility (potentiality) through which divine intelligence—or simply life and its functioning—creatively manifests itself in its effective organization of the world.

This specific activity that occurs in the *hypodoché* may be interpreted in various ways. We propose that the concept of the receptacle can serve as an original source of inspiration, making it possible to pose essential questions about the principles of our knowledge [4].

The idea of the receptacle embraces the universe as a very broad, not fully specified concept. Are there any indications that it holds true for some branches of science? We can competently speak about biology, which has become established on solid grounds thanks to genetics, and especially to more recent progress in molecular genetics [5]. After years of hesitation, it has been unequivocally accepted that deoxyribonucleic acid (DNA) represents the carrier of genetic information and codes for messenger RNA (mRNA), which directs the precise order of amino acid incorporation into the protein molecule (proteosynthesis). Such a principle does not exist *in vacuo* and is realized through many additional cell functions and subdued to epigenetic modifications.

At first glance, isolated DNA looks like amorphous slime. Not surprisingly, DNA construction has not been visible before resolving its primary structure and complementarity among nucleotides, which ensures DNA replication and its transcription to RNA. Only afterwards did we learn about the complex hierarchy of structural features, starting from secondary structures and DNA-protein interactions and ending with the chromatin organization and its nuclear architecture. In parallel, we learned about the complexity of recording genetic information in the form of genes, regulatory sequences, and long-distance interactions among all parts of what we today call the genome. Does DNA fit into the category of the receptacle previewed by Plato? There are some features that agree with such a view. The principles of DNA-mediated coding of RNA are kept in every known organism [6]. The three-letter code comprising two nucleotides is most general, but it might have evolved from a simpler two-letter code because the third letter is not fixed in most codons (codon wobbling). Like every other biological process, the genetic code is subject to evolutionary changes. Unambiguous three-letter coding of single amino acids is kept only in rare cases. Often, several codons are endowed with the ability to incorporate the same amino acid. This might be the result of codon mutation advantageous for the organism evolution. However, of great importance is codon reading mediated by the transfer RNA (tRNA) anticodon, which pairs with the codon [7]. Moreover, mutations in tRNA responsible for altered codon reading have been recognized [8]. Thus, we have in play not only the codon structure but also different ways of its reading following the demand for the most efficient reading and adjustment to unexpected new environmental challenges and to the mode of translation.

We also face the problem concerning the origin of the genetic code, which, in addition to the origin of life, remains enigmatic. It might have been originally represented by RNA, which is more flexible than DNA, and its structure can be more easily reshaped. From the virus family to which human immunodeficiency virus-causing acquired immunodeficiency syndrome belongs, we know the enzyme that can—in a reverse way—transcribe RNA to DNA. In addition, most viruses harbor RNA as their only genetic information. Thus, not knowing anything about the origin of life and early genetic information, we have in our hands extensive evidence on the evolution continuity of the genetic code.

Does this continuity have some relation to the Platonic receptacle? In order to fulfil this criterion, it should cope with inferences of other elements (water, air, fire/heat, earth) surrounding it and sharing its nature. From evolutionary biology, we have learned that the essential functions covering the metabolism, respiration, and others have common roots in known organisms. However, many other functions such as those related to gene expression, cell and tissue differentiation, and signal transduction from the cell membrane to genes are subject to significant variation. In this way, the genetic make-up reacts—with quite a delay—to exposure to different elements that correspond with the original receptacle proposal. Is the genome wobbly not only for exogenous, but also for some endogenous reasons? The answer has come more recently, based on the discoveries of several modes of how the genome can be and is rebuilt for internal causes. The finding that only about 1–2% of the genome is responsible for coding cell functions opened the question about what represents the remaining 98%. According to more recent investigations, these non-coding genome regions play an important role in gene regulation. Tight and complex regulation is a prerequisite for a relatively low number of constitutive gene adjustments to new challenges in the course of evolution. Moreover, about half of non-coding sequences arose by reverse transcription of RNA to DNA, which is made possible by specific enzymatic activity of some retrovirus-like elements that in themselves constitute a significant cell genome portion. Such elements exert the ability to move in the genome and alter its functions. Not surprisingly, these activities, on one hand, can be fatal and, on the other hand, can contribute to the evolution of new functions that remain, to a certain extent, under cellular control. There are multiple examples demonstrating exaptation of such elements or regulatory rewiring of the genomes by *de novo* insertions. Do such genome reshufflings contradict the idea of the receptacle? According to Plato’s postulation, *hypodoché*, by its motion, agitates even Empedocles’ elements.

If we accept Whitehead’s argument based on a thorough study of Plato’s works that ideas arise in connection with the traditional custom to clarify, and that ideas peak when new methods and institutions are established, we can only admire Plato’s prophecy. We are aware of a greater complexity of his *hypodoché*, which cannot simply be reduced to the genome composition and regulation, at least as far as we know. On the other hand, our genome represents an outcome of about three billion years of evolution and significantly influences the array of our capabilities. Although general ideas are unreachable and remain outside our present understanding, some of their aspects can be grasped by present-day scientific knowledge. Here, we have tried to present such an example.
